# Michigan State Board of Health

**Published:** 1896-06

**Authors:** 


### MICHIGAN STATE BOARD OF HEALTH.

A regular meeting was held at Lansing, April 10, 1896. The meeting was called to order by the President, Hon. Frank Wells, of Lansing, and the following members were present: Prof. Delos Fall, Albion; Dr. Samuel G. Milner, Grand Rapids; Dr. George H. Granger, Bay City; Judge Aaron V. McAlvay, Manistee; and Secretary Henry B. Baker, of Lansing. The regular business of auditing of bills and accounts was transacted.

The Board voted to direct the Secretary to request health-officers from whom no annual reports had been received for the year 1895 to make such reports immediately, in accordance with State law, or proceedings would be commenced against such delinquent officers. Reports by the Attorney-General, Judge McAlvay, and the Secretary of the Board, who read a letter from Health-officer Duffield, showed that the report of the health-officer of Detroit is being made out and may be expected in a few days.

One of the most important and interesting subjects which came to the attention of the Board was in connection with a communication from Governor John T. Rich, which suggested that the State Board of Health communicate with the proper official at each State institution in Michigan, especially the several asylums for the insane, calling attention to the prevalence of consumption in animals and in man, the danger of this disease being spread from animals to man by means of the milk-supply, and suggesting a plan whereby each institution could Pasteurize or in some way sterilize all the milk used. The Secretary mentioned that the subject of Pasteurization of milk was being taught at the Agricultural Experiment Station at Madison, Wis. If necessary, some person might be sent to this school for instruction who could return and teach the subject in this State. He mentioned that Mr. Grosvenor is said to be Pasteurizing milk for sale in Monroe, Michigan, and that a company in Northville has been thus preparing large quantities of milk for sale in Detroit. The Board directed the Secretary to send to the several State institutions communications which shall cover the Governor's suggestions for the sterilization of the milk-supply for the inmates of State institutions.

In connection with this subject, the Secretary read an item relative to a farmer who lost two head of cattle from tuberculosis. Later the disease developed in the farmer's family, consisting of six members, all of whom, together with two attendants, have since died with consumption.

The difficulties in the way of inducing the people generally to act on this subject are many, but the Board recognizes the fact that it is of very much greater importance that the milk-supplies of cities and villages shall be sterilized so as to be free from tuberculosis and typhoid-fever germs than it is to protect only the inmates of State institutions. The Board directed its Secretary to prepare for publication and general distribution a forcible statement of the facts and dangers from infected milk, and methods for the sterilization of the public and domestic milk-supplies.

The Board considered an informal invitation for a sanitary convention at Belding, Michigan. It was voted that the Secretary should correspond with parties in Belding stating that the Board would hold a sanitary convention, providing the citizens of that city would insure that every effort would be made to make the meeting a success.

It was also decided to hold another of the popular conferences of Michigan health-officers at Ann Arbor. The Secretary was requested to correspond with the director of the State Laboratory of Hygiene, at Ann Arbor, regarding the time that such a meeting could best be held.

The Secretary presented a complete report of the work of the office during the last quarter. The report showed that in consequence of new laws the work of the office was greater than formerly, but that it is being brought up to date; and the work required by the new school-law seems to be doing a great amount of good.

The Secretary made a special report upon the distribution of the Board's leaflets and pamphlets relative to communicable diseases, to school-teachers, county school commissioners, city school superintendents, and in a number of instances to pupils in high schools. This report showed that there are about 16,000 teachers in Michigan, and that about 20,000 sets of such publications had been sent out. This distribution of leaflets was in compliance with Act 146, laws of 1895, which requires the Board to supply data and statements bearing upon the modes of spreading and the best methods for the restriction and prevention of the dangerous communicable diseases, which subject is required to be taught in every public school in Michigan.

